# Fluorodeoxyglucose-positron emission tomography/computed tomography in the staging and evaluation of treatment response in a patient with Castleman's disease: a case report

**DOI:** 10.1186/1752-1947-2-99

**Published:** 2008-04-03

**Authors:** Ettore Pelosi, Andrea Skanjeti, Angelina Cistaro, Vincenzo Arena

**Affiliations:** 1 IRMET PET Center, Via Onorato Vigliani, 10138 Turin, Italy; 2 Nuclear Medicine Unit, University of Turin, Corso Bramante, 10126 Turin, Italy

## Abstract

**Introduction:**

Castleman's disease is a rare lymphatic polyclonal disorder that is characterised by unicentric or multicentric lymph node hyperplasia and non-specific symptoms and signs including fever, asthenia, weight loss, enlarged liver and abnormally high blood levels of antibodies.

**Case presentation:**

We present the case of a 74-year-old man with Castleman's disease. The disease was detected with a contrast-enhanced computed tomography (CT) scan and a fluorodeoxyglucose (FDG)-positron emission tomography (PET)/CT study; diagnosis was made with histopathology. After treatment with surgical excision followed by chemotherapy, the disease response was evaluated using both diagnostic techniques. However, only the PET study was able to identify the spread of the disease to the abdominal lymph nodes, which were both enlarged and normal size, and, after treatment, to evaluate the disease response.

**Conclusion:**

Based on the results of previous case reports and on those of the present study, it seems that Castleman's disease has a high glucose metabolic activity. Therefore, the use of PET can be considered appropriate in order to stage or restage the disease and to evaluate the response of the disease to treatment.

## Introduction

Castleman's disease is a rare lymphatic polyclonal disorder that is characterised by unicentric or multicentric lymph node hyperplasia and non-specific symptoms and signs including fever, asthenia, weight loss, enlarged liver and abnormally high blood levels of antibodies. In 1954, Castleman and Towne [1] described the first case of the disease in a patient with a mediastinal mass. Then, other authors reported new cases of the disease with different localisations, including abdominal and superficial lymph nodes. The aetiology and pathogenesis are still unclear and under debate. Diagnosis and classification are based on histopathological analysis. Surgical excision is the recommended treatment in the unicentric form, while different systemic therapeutic strategies can be adopted for the multicentric form [2].

## Case presentation

A 74-year-old man was referred to our centre in July 2006, for a mesenterial lymphatic mass to be characterised metabolically with 18F fluorodeoxyglucose (FDG)-positron emission tomography (PET). The patient was treated previously for prostatic adenocarcinoma with radiotherapy and anti-androgen treatment with bicalutamide. During the follow-up, an anomalous lymph node (size 32 mm × 50 mm) at the mesenterial level was identified using both ultrasonographic examination and a contrast-enhanced computed tomography (CT) scan.

**Figure 1:**
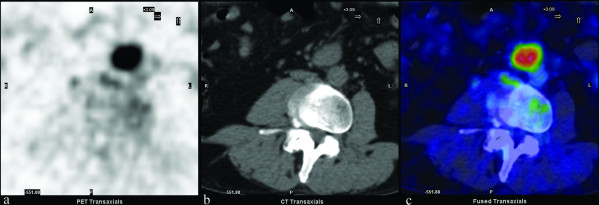
**FDG-PET/CT transaxial views of the mesenterial lesion.** (A) PET; (B) low-dose CT; (C) PET/CT fusion.

**Figure 2:**
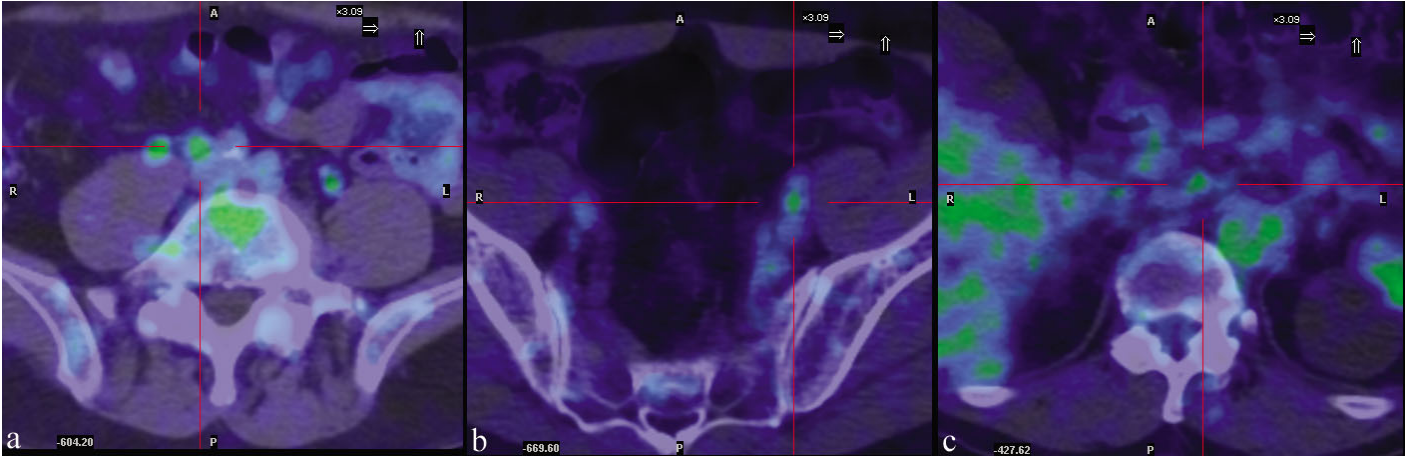
**FDG-PET/CT transaxial views (PET/CT fusion).** (a), (b) Iliac bilateral and (c) mesenteric pathologic lymph nodes.

The PET scan was acquired 60 minutes after intravenous injection of FDG (248 MBq). At the time of the tracer injection, the patient had fasted for more than six hours, and the glucose blood level was 81 mg/dl. The PET study showed the presence of anomalous tracer uptake in the mesenterial mass (maximum standardised uptake value [SUVmax] 6.03; Figure 1). Further pathological tracer uptakes were depicted in the lymph nodes (not exceeding 15 mm in size) in the mesenteric and iliac bilateral regions (Figure 2).

**Figure 3:**
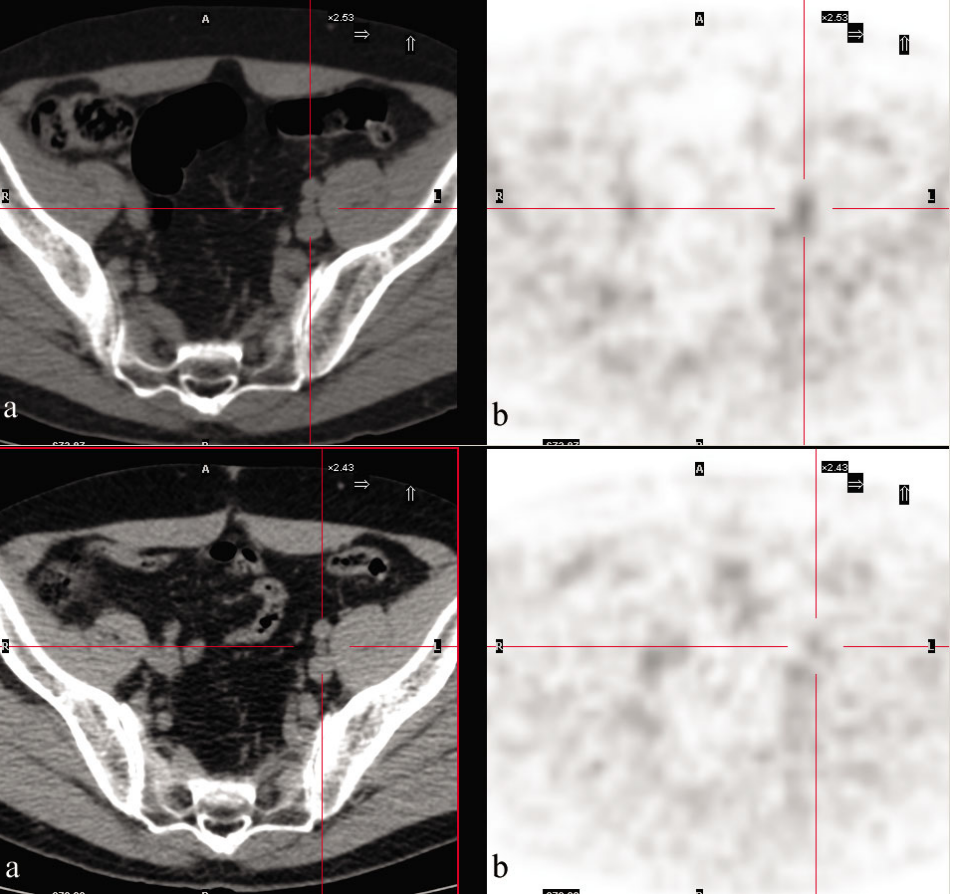
**FDG-PET/CT transaxial views of the left iliac lymph node.** (A) Low-dose CT; (B) PET. Upper images: pre-treatment (SUVmax 2.4); lower images: post-treatment (SUVmax 1.8).

Based on this result, the patient underwent excision of the mesenterial lymphatic mass and histopathological analysis showed Castleman's disease of the hyalin-vascular subtype. From October 2006 to February 2007, the patient was treated with four cycles of chemotherapy. A further CT scan of the abdomen was performed at the end of the treatment and then again in June 2007; a second PET/CT study with FDG was performed in June 2007. The CT scan showed the persistence of the mesenteric and iliac lymph nodes which remained stable in size. However, FDG-PET scan did not reveal any pathological uptake in those lymph nodes suggesting a complete disease response to the treatment (Figure 3).

No clinical signs suggestive of recurrence were seen during a follow-up at five months.

## Discussion

FDG-PET/CT is a hybrid diagnostic technique used in many neoplasms and aggressive malignant lymphomas to characterise metabolically undetermined masses, tumour staging and restaging, treatment response evaluation and radiotherapy treatment planning. In fact, it allows a combination of both anatomical and biological co-registered images acquired in the same session, with a dual gain in diagnostic accuracy.

Staging is crucial in the identification of the appropriate treatment in Castleman's disease. CT or magnetic resonance imaging (MRI) is commonly used. The usefulness of FDG-PET/CT has been reported in a few cases [3-7]. However, based on the results of these reports and on those of the present study, PET seems to represent the most appropriate approach. In fact, Castleman's disease, as with aggressive lymphomas and many solid tumours, presents an increase in glucose metabolic activity. Therefore, PET study can lead to a more precise staging of the disorder since the disease can be present in normal-sized lymph nodes as in our case and, alternatively, reactive lymph nodes with increased size can be erroneously judged as pathological. Furthermore, a PET study can be used to evaluate disease response to treatment as in our case: although the lymph nodes were still present after treatment, their metabolic activity had significantly decreased suggesting, together with clinical signs, a complete disease response.

## Conclusion

This case report shows that FDG-PET/CT could have an important role in the staging of Castleman's disease and in its evaluation of treatment response.

## Competing interests

The author(s) declare that they have no competing interests.

## Authors' contributions

EP is the senior author and was involved in collecting patient details, reviewing the literature and final proofreading of the manuscript. AS was involved in collecting patient details, reviewing the literature and drafting the manuscript. AC was involved in reviewing the literature and proofreading the manuscript. VA approved the final manuscript.

## Consent

Written informed consent was obtained from the patient for publication of this case report and accompanying images. A copy of the written consent is available for review by the Editor-in-Chief of this journal.
